# Identification and validation of pivotal genes related to age-related meniscus degeneration based on gene expression profiling analysis and in vivo and in vitro models detection

**DOI:** 10.1186/s12920-021-01088-6

**Published:** 2021-09-29

**Authors:** Ming Chen, Siqi Zhou, Huasong Shi, Hanwen Gu, Yinxian Wen, Liaobin Chen

**Affiliations:** 1 Division of Joint Surgery and Sports Medicine, Department of Orthopedics Surgery, Zhongnan Hospital of Wuhan University, Wuhan, 430071 China; 2 Hubei Provincial Key Laboratory of Developmentally Originated Disease, Wuhan, 430071 China; 3 Department of Orthopedics Surgery, Renmin Hospital of Wuhan University, Wuhan, 430060 China; 4 Joint Disease Research Center, Wuhan University, Wuhan, 430071 China

**Keywords:** Meniscus degeneration, Bioinformatics analysis, Senescence

## Abstract

**Background:**

The componential and structural change in the meniscus with aging would increase the tissue vulnerability of the meniscus, which would induce meniscus tearing. Here, we investigated the molecular mechanism of age-related meniscus degeneration with gene expression profiling analysis, and validate pivotal genes in vivo and in vitro models.

**Methods:**

The GSE45233 dataset, including 6 elderly meniscus samples and 6 younger meniscus samples, was downloaded from the Gene Expression Omnibus (GEO) database. To screen the differential expression of mRNAs and identify the miRNAs targeting hub genes, we completed a series of bioinformatics analyses, including functional and pathway enrichment, protein–protein interaction network, hub genes screening, and construction of a lncRNA–miRNA–mRNA network. Furthermore, crucial genes were examined in human senescent menisci, mouse senescent meniscus tissues and mouse meniscus cells stimulated by IL-1β.

**Results:**

In total, the most significant 4 hub genes (*RRM2, AURKB, CDK1,* and *TIMP1*) and 5 miRNAs (hsa-miR-6810-5p, hsa-miR-4676-5p, hsa-miR-6877-5p, hsa-miR-8085, and hsa-miR-6133) that regulated such 4 hub genes, were finally identified. Moreover, these hub genes were decreased in meniscus cells in vitro and meniscus tissues in vivo, which indicated that hub genes were related to meniscus senescence and could serve as potential biomarkers for age-related meniscus tearing.

**Conclusions:**

In short, the integrated analysis of gene expression profile, co-expression network, and models detection identified pivotal genes, which elucidated the possible molecular basis underlying the senescence meniscus and also provided prognosis clues for early-onset age-related meniscus tearing.

## Introduction

The meniscus is an important structure of knee joint, playing a vital role in load transmission, shock absorption, joint lubrication and nutrition, proprioception, and stability [1]. The position of the meniscus and its function in load transfer and shock absorption make the meniscus prone to traumatic and degenerative injuries. Therefore, the meniscus tearing is one of the most common intra-articular injuries of knee joint, and also one of the major causes of gonyalgia and lameness [2, 3]. Other studies have also suggested that meniscus tearing injury is not only one of the high-risk factors for inducing osteoarthritis (OA), but also further aggravates the process of osteoarthritis, and ultimately affects people's basic daily activities [4, 5].

The population-based cohort study found that the age is a major risk factor for meniscus tearing, especially in people over 40 years old [6, 7]. Previous studies have shown that aging could cause compositional changes in the meniscus, which includes degeneration of collagens, cells, and proteoglycans [8, 9]. Meanwhile, these age-related tissue changes, such as aggregation of advanced glycation end products, thickening of collagen fibers, and mucoid degeneration, would increase the tissue vulnerability of the meniscus, which would eventually induce meniscus tearing [10]. Tsujii et al. found that age-related tissue homeostasis changes in the meniscus might be caused by senescence of meniscus cells [8]. However, there are few studies on meniscus aging, and also the specific molecular mechanism and key genes of meniscus cell senescence are still unclear [11, 12].

Bioinformatics is an emerging interdisciplinary field which combines molecular biology and information technology. It is of great importance to reveal that the differentially expressed genes (DEGs) are involved in molecular mechanisms, specific pathways, protein–protein interactions (PPI), and associations between disease and genes [13]. In order to study the molecular mechanism of age-related meniscus degeneration and further explore potential biological therapeutic targets, we carried out extensive bioinformatics methods to identify key DEGs and functional pathways in the aged meniscus tissues. Then, we constructed a lncRNA-miRNA-mRNA network to search potential miRNAs that may act on the screened key DEGs. Moreover, we further confirmed the expressions of these key DEGs in human senescent menisci, mouse aging meniscus tissues, and IL-1β-stimulated mouse meniscus cells.

## Materials and methods

### Chemicals and reagents

IL-1β was purchased from PeproTech Co., Ltd (Rocky, USA). The dulbecco's modified eagle medium-F12 (DMEM/F12) and phosphate buffer saline (PBS) were obtained from HyClone Co. (Logan, USA), while fetal bovine serum (FBS) was ordered from Gibco Co. (Detroit, USA). The TRIzol reagent was purchased from Invitrogen Co. (Carlsbad, USA). The SYBR Green dye and reverse transcription kits were purchased from Servicebio Co., Ltd (Wuhan, China). The antibodies for ribonucleotide reductase regulatory subunit M2 (*RRM2*, A5255), tissue inhibitor of metalloproteinases 1(*TIMP1*, A1389), and cyclin dependent kinase 1(*CDK1*, A0220) were ordered from ABclonal Technology Co., Ltd. (Wuhan, China). The aurora kinase B (*AURKB*, ab2254) and tissue inhibitor of metalloproteinases 1(*TIMP1*, ab216432) were purchased from Abcam plc. (Cambridge, UK). The SA-β-gal staining kits were obtained from Beyotime Co., Ltd. (Shanghai, China). The Annexin V-FITC/7-ADD apoptosis detection kits (Catalog Number: APK10448-F) were ordered from Sino Biological Co., Ltd. (Beijing, China). All primers were Tianyi Biotech Co., Ltd. (Wuhan, China).

### Data collection

The GSE45233 dataset in Gene Expression Omnibus was downloaded for subsequent analysis. GSE45233 based on GPL10558 platform (Illumina HumanHT-12 V4.0 expression beadchip; Microarrays, Inc., San Diego, CA, USA) was demarcated by the age of 40, including 6 elderly meniscus samples and 6 younger meniscus samples, which were obtained from patients undergoing arthroscopic partial meniscectomy [14, 15].

### Identification of DEGs in elderly and younger samples

We used the robust multiarray average algorithm to perform background correction and quartile data normalization on the GSE45233 dataset, and then identified DEGs associated with age by means of the Limma package in R language (version3.6.3). *P* value < 0.05 and log_2_ fold change (log_2_FC) <  − 1.5 or log_2_FC > 1.5 were defined as the cutoff standard. The volcano plot and heatmap were completed in virtue of Ggplot2 and Pheatmap packages.

### KEGG and GO analysis annotate the functions of DEGs

DAVID (version 6.8; https://david.ncifcrf.gov/) is an online bioinformatics database that integrates biological data and analysis tools to provide systematic and comprehensive biological annotation information for extensive lists of genes and proteins. The concept of Gene Ontology (GO) is widely used in the field of bioinformatics, which contains three notions of functional information: biological processes (BP), cellular components (CC), and molecular functions (MF). Currently, Kyoto Encyclopedia of Genes and Genomes (KEGG; https://www.kegg.jp/) is a database that integrates genomic, chemical, and system functional information for signaling pathway analysis. We employed the DAVID online tool to perform GO and KEGG analysis of DEGs.

### Construction of the PPI network

STRING (Version11.0; https://string-db.org/), a search tool for interacting genes/proteins, was applied for predicting the PPI network and discovering the possible relationships. Cytoscape (version 3.8.0; https://cytoscape.org/), a powerful network building software, was used to establish the PPI network.

### Identification and analysis of significant hub genes

The hub genes were screened through the PPI network utilizing the Cytoscape plugin cytoHubba. Since the singlular algorithm is sometimes biased, we employed a four-fold algorithm to collectively recognize hub genes, and these algorithms were adopted respectively. The algorithms used to recognize hub genes include Edge Percolated Component (EPC), Degree, Maximal Clique Centrality (MCC), and Maximum Neighborhood Component (MNC). Then the common hub genes were obtained through the intersection of Venn diagram websites (http://bioinformatics.psb.ugent.be/webtools/Venn). Metascape, as a forceful gene function analysis and annotation tools, was applied for batch enrichment analysis and network construction of genes and proteins to understand their functions. The common hub genes were further analysed through Metascape analysis.

### Construction of lncRNA–miRNA–mRNA network

miRWalk (http://mirwalk.umm.uni-heidelberg.de/), as a bioinformatics suit to forecast miRNA–mRNA interactions, could predict miRNAs of the common Hub gene and then obtain the miRNA–mRNA interaction pairs with the score > 0.9 [16]. The DIANA-miRPath (https://www.microrna.gr/miRPathv3) is an online tool dedicated to assessing the regulatory role of miRNAs and forecasting relevant regulation pathways. The DIANA-LncBase (http://carolina.imis.athena-innovation.gr/diana_tools/) was conducted to search the lncRNAs targeting at miRNAs. In the end, a lncRNA–miRNA–mRNA regulatory network was accomplished by means of Cytoscape software.

### Human/mouse meniscus tissues from normal and aging knees

Human meniscus tissues were obtained from 12 patients undergoing arthroscopic partial meniscectomy, including 6 patients older than 40 years and 6 patients younger than 40 years. This human research protocol was authorized by the Medical Ethics Committees (approval number: 2019K-K011). All patients have signed informed consent. Animal protocols used in this study were approved by the Animal Welfare Committee (License number: 14016). Mouse meniscus tissues were collected from specific pathogen free (SPF) C57BL/6 J male mice [NO.2020-0018, license number: SCXK (Hubei), certification number:42000600040335] purchasing from the Experimental Center of the Hubei Medical Scientific Academy (Wuhan, China) of two different ages, namely 3 months old (n = 6) and 18 months old (n = 6). All animals in this experiment were housed in a clean environment under a 12 h light/dark cycle for 1 week, where standard laboratory chow and water were freely consumed. Then, after the mice were anesthetized by intraperitoneal injection of 1% pentobarbital (60 mg/kg), the mice were euthanatized by spinal dislocation and the femurs were collected for subsequent detection.

### Histological analysis in the meniscus tissues

After fixation with 4% paraformaldehyde for 2 days, the entire mouse knee joints and human meniscus were decalcified, dehydrated and embedded in paraffin. The sections of mouse knee joints were stained with Safranin-O Fast Green and Toloniumchloride Blue to observe histopathological changes.

For immunohistochemistry analysis, knee joint sections were deparaffinized, hydrated, antigen retrieval, and blocked in 3% BSA (Servicebio, Wuhan, China) at room temperature for 1 h. Then, the sections incubated with primary antibodies for *AURKB* (1:200, dilution), *CDK1* (1:100, dilution), *RRM2* (1:100, dilution), and *TIMP1*(1:250, dilution, Abcam) overnight at 4 °C, followed by incubating with biotinylated secondary antibody (1:100 dilution). Finally, after the sections incubating with an avidin‐biotinylated HRP complex solution, these were immersed in diaminobenzidine (DAB) to reveal peroxidase activity. Observations and photographs were taken under the Photo Imaging System (Nikon H550S, Japan). For immunohistochemical-negative controls, the primary antibodies were replaced by non-immunized IgG in immunostaining. Then, we measured the mean optical density (MOD) of 5 random fields in each section using Image Pro-plus (version 6.0) to determine the dyeing intensity. The percentage of positive cells in each field was the ratio of the number of positive cells to the total number of meniscus cells in the corresponding region.

### SA-β-gal staining

The mouse meniscus cells were derived from meniscus tissues of SPF 3-month-old C57BL/6J mice. In brief, the full thickness meniscus tissues were cut into pieces, and digested with serum-free DMEM/antibiotics containing collagenase D (Roche, Basel, Switzerland) for 6 h at 37 °C [17]. The meniscal cells were cultured on DMEM containing 10% FBS and inoculated in 12-well plates. Then, treatment of the cells was conducted with IL-1β (10 ng/ml) for 2d to achieve the induction of cell senescence injury by inflammatory factors [18]. The meniscus cells were fixed at room temperature for 15 min, after washing three times with PBS, and stained with β-galactosidase staining solution for 12 h at 37 °C without CO_2_ environment. After that, these were rinsed again with PBS and were observed via an inverted microscope (Nikon, Japan).

### Apoptosis assay

For apoptosis analysis, the meniscus cells treated with IL-1β (10 ng/ml) for 2 days. After cells resuspended with 100 μL1x binding buffer, 5 μL Annexin V-FITC and 5 μL 7-ADD were added and incubated at room temperature for 15 min without light. The apoptosis rate was detected by using a FACS Aria III Flow cytometry (BD Biosciences, USA).

### Cell immunofluorescence analysis

For immunofluorescence staining, the meniscus cells were treated with IL-1β (10 ng/ml) for 2 days, and then the cells were fixed in 4% paraformaldehyde for 15 min at room temperature. After washing with PBS, the cells were permeabilized with 0.3% Triton X-100 for 15 min and blocked with 3%BSA at room temperature for 40 min. Then, the cells were washed with PBS and incubated with primary antibodies of *CDK1*(1:200, dilution), *AURKB* (1:200, dilution), *RRM2* (1:100, dilution), and *TIMP1*(1:150, dilution, Abclonal) at 4℃ overnight. The meniscus cells were rinsed three times with PBS and incubated with secondary antibodies (1:100; ThermoFisher, USA) for 1 h at room temperature the next day. In the end, we stained the nuclei with 4',6-diamidino-2-phenylindole (DAPI, Thermo Fisher, USA) for 5 min. All of the images were captured under the fluorescence microscope (Nikon, Japan).

### qRT-PCR confirmation of the hub genes

Total RNA from meniscus tissue was extracted with TRIzol reagent following the manufacturer's protocol. After the purity and concentration of isolated RNA were determined by the nano-drop-2000 nucleic acid analyzer (ThermoFisher, USA), the RNA reverse transcription kits were devoted to reverse transcribe total RNA into cDNA. Quantitative real-time PCR (qRT-PCR) was performed with the ABI StepOnePlus cycler (Applied Biosystems, USA) in a 10-μl reaction system. To accurately quantify the gene expression levels, mRNA levels of the internal reference gene glyceraldehyde phosphate dehydrogenase (*GAPDH*) was normalized by the 2^−ΔΔCT^ method. The primer sequences of genes applied in this study were listed in the Table 1.

**Table 1:** The primer sequences of genes in this experiment

Target genes	Species	Forward primer	Reverse primer
*RRM2*	Homo	GTTTGTGGCAGACAGACTTA	TCACTCCCATCCTCTGATAC
*RRM2*	Mouse	TAGGCGAGTATCAGAGGATG	GTGTAGCCAGTTGGTTGTT
*AURKB*	Homo	CCAGAAGGTGATGGAGAATAG	CCCATGGCAGTACATTAGAG
*AURKB*	Mouse	CTACAAGGAACTGCAGAAGAG	CAGGTTCTCCGGCTTTATG
*CDK1*	Homo	GTCAGCTCGTTACTCAACTC	CTTCTGGCCACACTTCATTA
*CDK1*	Mouse	CATGGTCAGAGGTAGGTTAGA	CTAAGCAGCACAGCGATAC
*TIMP1*	Homo	CCACCTTATACCAGCGTTATG	CAGGTAGTGATGTGCAAGAG
*TIMP1*	Mouse	GGCATCCTCTTGTTGCTATC	GGTGGTCTCGTTGATTTCTG
*GAPDH*	Homo	GAAGGTGAAGGTCGGAGTC	GAAGATGGTGATGGGATTTC
*GAPDH*	Mouse	AGGTCGGTGTGAACGGATTTG	TGTAGACCATGTAGTTGAGGTCA

*RRM2* ribonucleotide reductase regulatory subunit M2; *AURKB* aurora kinase B; *CDK1* cyclin dependent kinase 1; *TIMP1* tissue inhibitor of metalloproteinases 1; *GAPDH* glyceraldehyde phosphate dehydrogenase

### Statistical analysis

The expression levels of hub genes that were differentially expressed between young and aging tissues or cells were determined by implementing Student’s *t*-test with Prism 6.0 (GraphPad Software, USA), and the data of the experimental results were presented as mean ± standard error of mean (SEM). In addition, for GO functional annotation, DAVID calculated the *P* value of the false discovery and enrichment rate via Benjamini correction. The *P* < 0.05 was considered to be statistically significant difference.

## Results

### Identification of DEGs

**Fig. 1:**
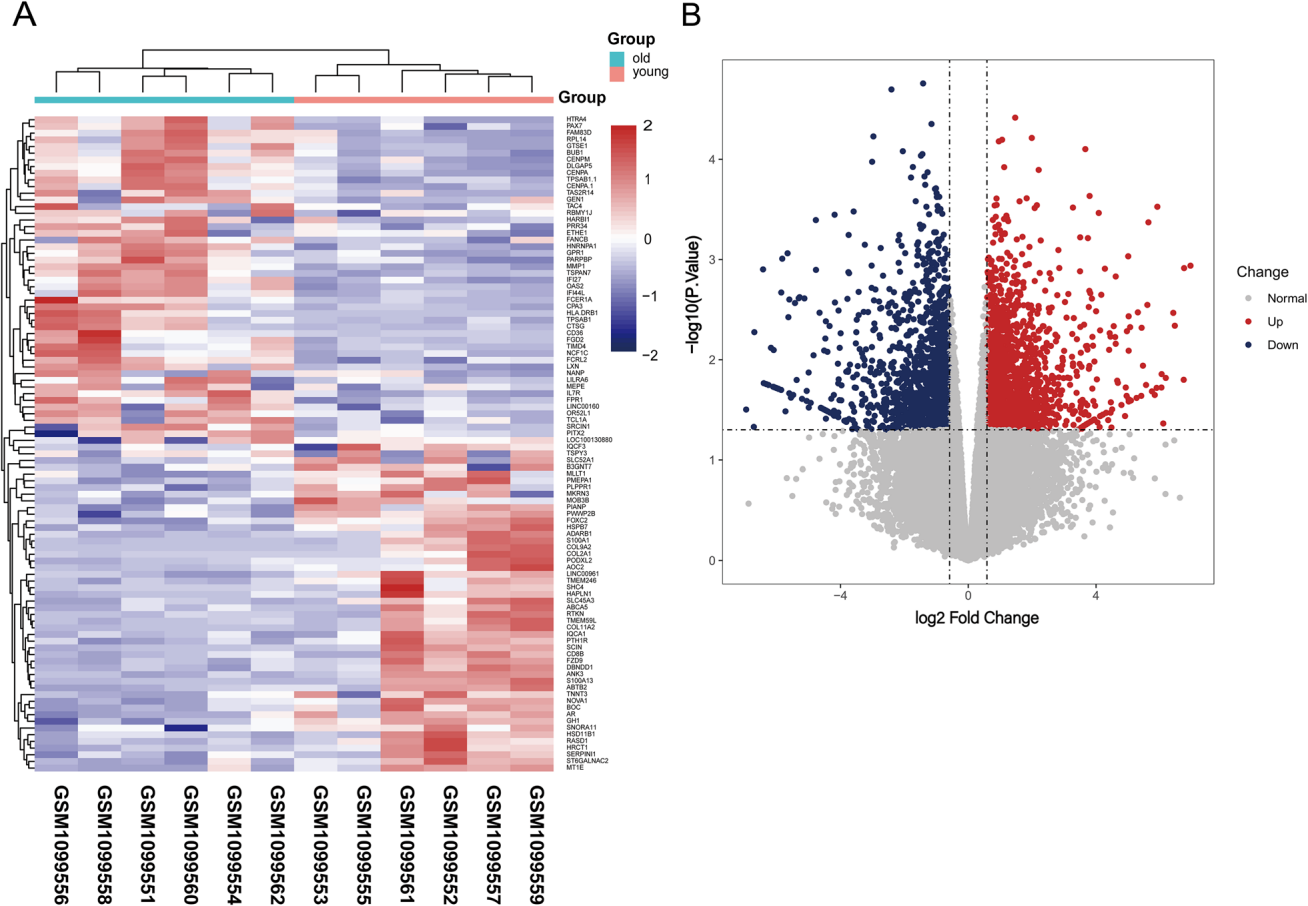
The heatmap and volcano plot of differentially expressed genes (DEGs) between gerontic and younger groups. **A** DEGs expression heatmap of meniscus tissues. **B** DEGs was screened by volcano map filtering method (*P*-value < 0.05 and | log_2_FC|> 1.5). “log2FC” means Log2 of the fold change

A total of 631 DEGs, including 341 up-regulated genes and 290 down-regulated genes, were identified from the GSE45233 dataset by comparing the gerontic and young meniscus samples. The heatmap and volcano plot of total DEGs were shown in Fig. 1A, B, respectively.

### GO function and KEGG pathway enrichment analysis of the DEGs

**Fig. 2:**
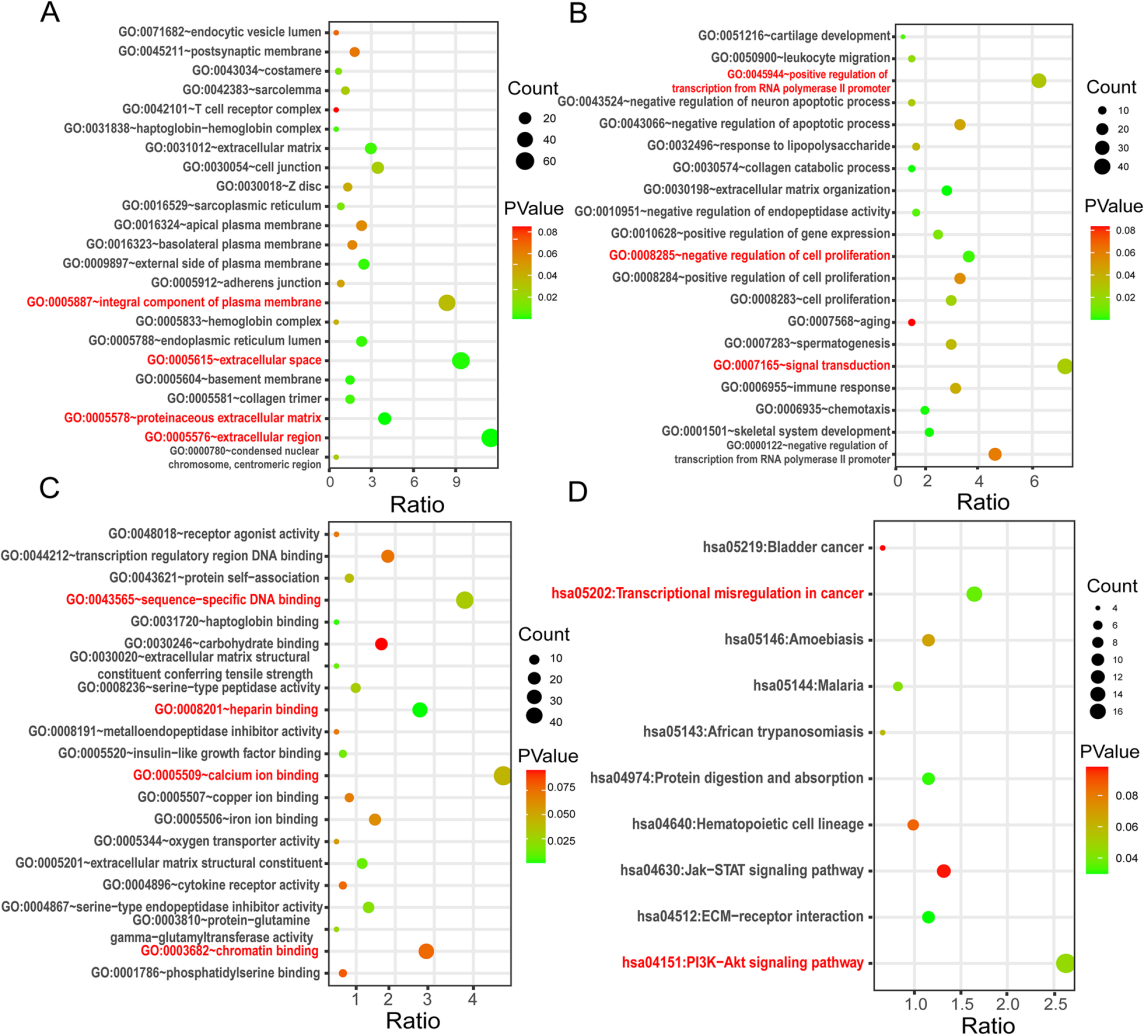
The functional enrichment analysis bubble diagrams of differentially expressed genes (DEGs) by DAVID. Detailed variations information bubble diagrams of GO categories: **A** CC, **B** BP, and **C** MF. **D** KEGG pathway analysis bubble diagram for DEGs. *GO* gene ontology; *CC* cellular component; *BP* biological processes; *MF* molecular functions; *KEGG* Kyoto Encyclopedia of Genes and Genomes

The results of the GO analysis showed that DEGs related to CC were notably concentrated in extracellular region, extracellular space, integral component of plasma membrane, and proteinaceous extracellular matrix (Fig. 2A). Variations in DEGs connected with BP were mainly concentrated in signal transduction, regulation of transcription from RNA polymerase II promoter, and regulation of cell proliferation (Fig. 2B). Regarding MF, DEGs were prominently gathered in calcium ion binding, chromatin binding, sequence-specific DNA binding, and heparin-binding (Fig. 2C). KEGG pathway analysis manifested that the top typical pathways correlated with DEGs were PI3K-Akt signaling pathway and transcriptional misregulation in cancers (Fig. 2D).

### Construction of PPI network and hub genes analysis

**Fig. 3:**
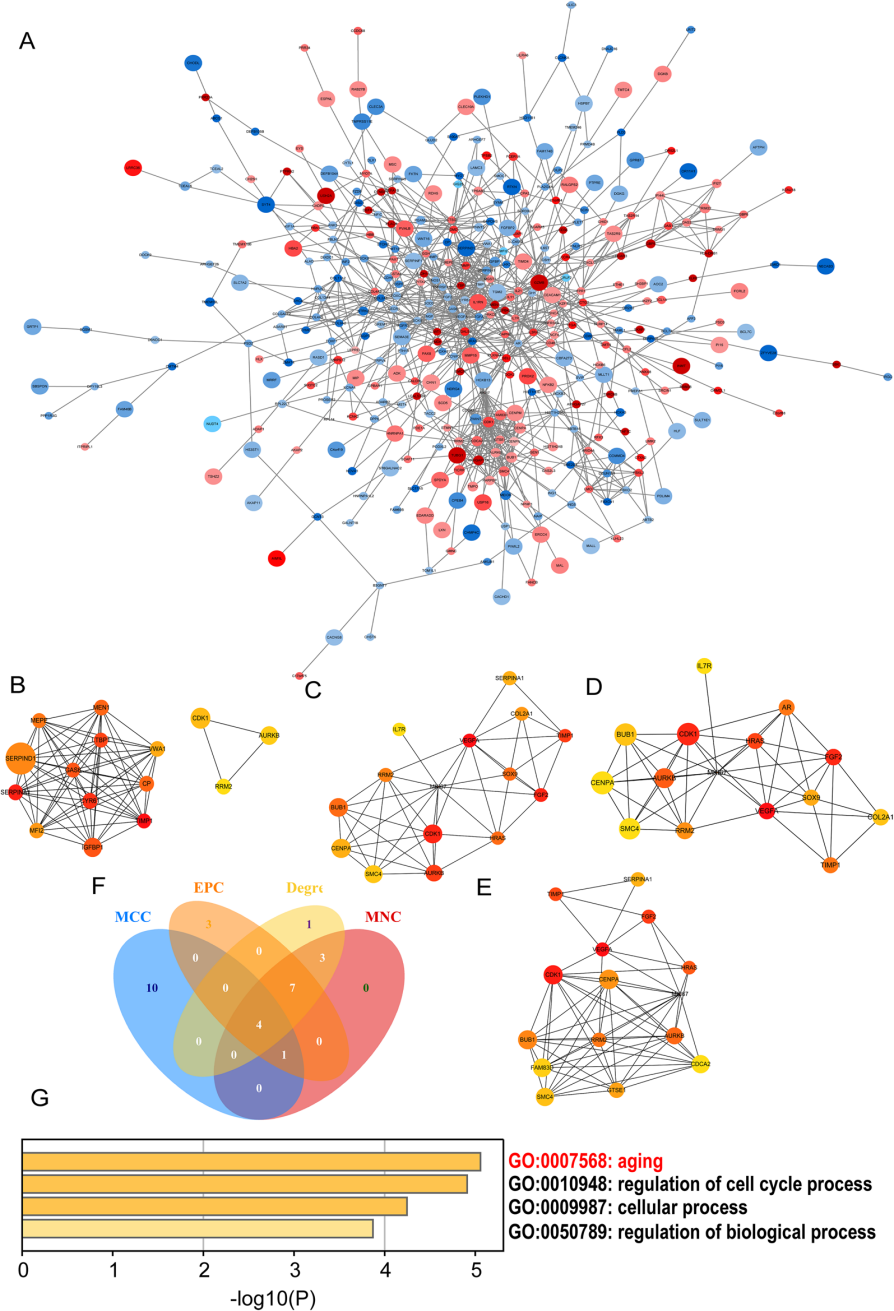
The PPI network and Hub genes analysis. **A** PPI network of all DEGs (The red codes represent upregulated genes in the elderly, the blue codes represent downregulated genes in the elderly, and the size of the nodes represents the correlation between them). Hub gene networks obtained from the PPI network relying on **B** the Maximal Clique Centrality (MCC) algorithm, **C** the Maximum Neighborhood Component (MNC) algorithm, **D** the Degree algorithm, and **E** the Edge Percolated Component (EPC) algorithm of the Cytoscape plugin cytoHubba. **F** The Venn diagram showed the identified hub genes (*RRM2, AURKB, CDK1,* and *TIMP1*). **G** The heatmap of common hub genes identified by Metascape was colored by –log_10_ (P-value). *PPI* protein–protein interaction; *DEGs* differentially expressed genes; *RRM2* ribonucleotide reductase regulatory subunit M2; *AURKB* aurora kinase B; *CDK1* cyclin dependent kinase 1; *TIMP1* tissue inhibitor of metalloproteinases 1

The PPI network of the DEGs was constructed by employing STRING (Fig. 3A). The Degree, MCC, MNC, and EPC algorithms were applied to filtrate hub genes shown in Fig. 3B–E, respectively. The mutual hub genes (*RRM2, AURKB, CDK1,* and *TIMP1*) were discerned by the Venn diagram (Fig. 3F). According to Metascape analysis, GO enrichment showed that the significant hub genes related to BP were mainly concentrated in aging and regulation of the cell cycle process (Fig. 3G).

### Construction of a lncRNA–miRNA–mRNA network

**Fig. 4:**
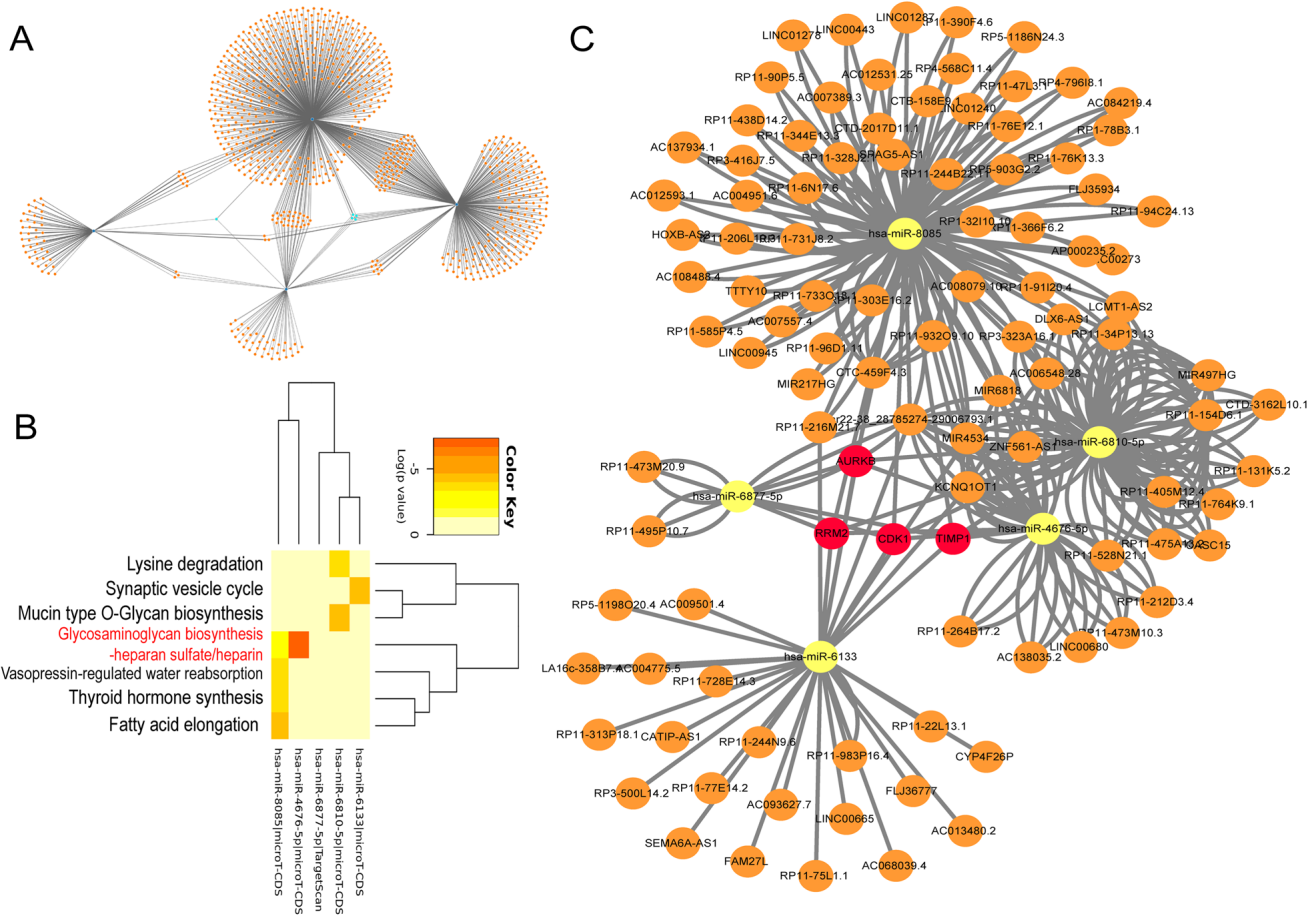
Network construction of lncRNA-miRNA-mRNA. **A** The mRNA-miRNA network was constructed by using the miRWalk website (The five miRNAs most associated with the common hub gene are highlighted in light blue). **B** Meaningful signal pathways about the 5 miRNAs by utilizing the DIANA-miRPath. **C** The lncRNA–miRNA–mRNA regulatory network (The orange nodes represent the lncRNAs, the yellow nodes represent key miRNAs, and the red nodes represent the hub genes). *RRM2* ribonucleotide reductase regulatory subunit M2; *AURKB* aurora kinase B; *CDK1* cyclin dependent kinase 1; *TIMP1* tissue inhibitor of metalloproteinases 1

As for the screening of miRNAs, since there were no miRNAs that could act on all Hub genes, miRNAs working on three Hub genes were selected as ideal targets. Through the miRWalk website predictive analysis, we screened 5 miRNAs (hsa-miR-6810-5p, hsa-miR-4676-5p, hsa-miR-6877-5p, hsa-miR-8085, and hsa-miR-6133) (Fig. 4A, Table 2). As illustrated in Fig. 4B, these miRNAs are referred to some pathways that include glycosaminoglycans biosynthesis. To further understand the function of the hub genes in meniscal degeneration, we employed Cytoscape to combine lncRNA/miRNA interactions with miRNA/mRNA interactions, thus constructing a lncRNA-miRNA-mRNA network (Fig. 4C).

**Table 2:** Information of the 5 miRNAs most relevant to the common hub genes

	Target genes	Position	Energy	Binding region length	*P*-value
hsa-miR-6810-5p	RRM2,CDK1,TIMP1	3UTR	− 24.1	29	0.01664477
hsa-miR-4676-5p	RRM2,CDK1,TIMP1	3UTR	− 22.9	18	0.007139029
hsa-miR-6877-5p	RRM2,CDK1,TIMP1	3UTR	− 21.8	24	0.000873123
hsa-miR-8085	RRM2,CDK1,TIMP1	3UTR	− 22.6	25	0.006883728
hsa-miR-6133	RRM2,AURKB,TIMP1	3UTR	− 22.4	17	0.001472693

*RRM2* ribonucleotide reductase regulatory subunit M2; *AURKB* aurora kinase B; *CDK1* cyclin dependent kinase 1; *TIMP1* tissue inhibitor of metalloproteinases 1

### The expression of AURKB, RRM2, CDK1, and TIMP1 were decreased in senescent meniscus tissues

**Fig. 5:**
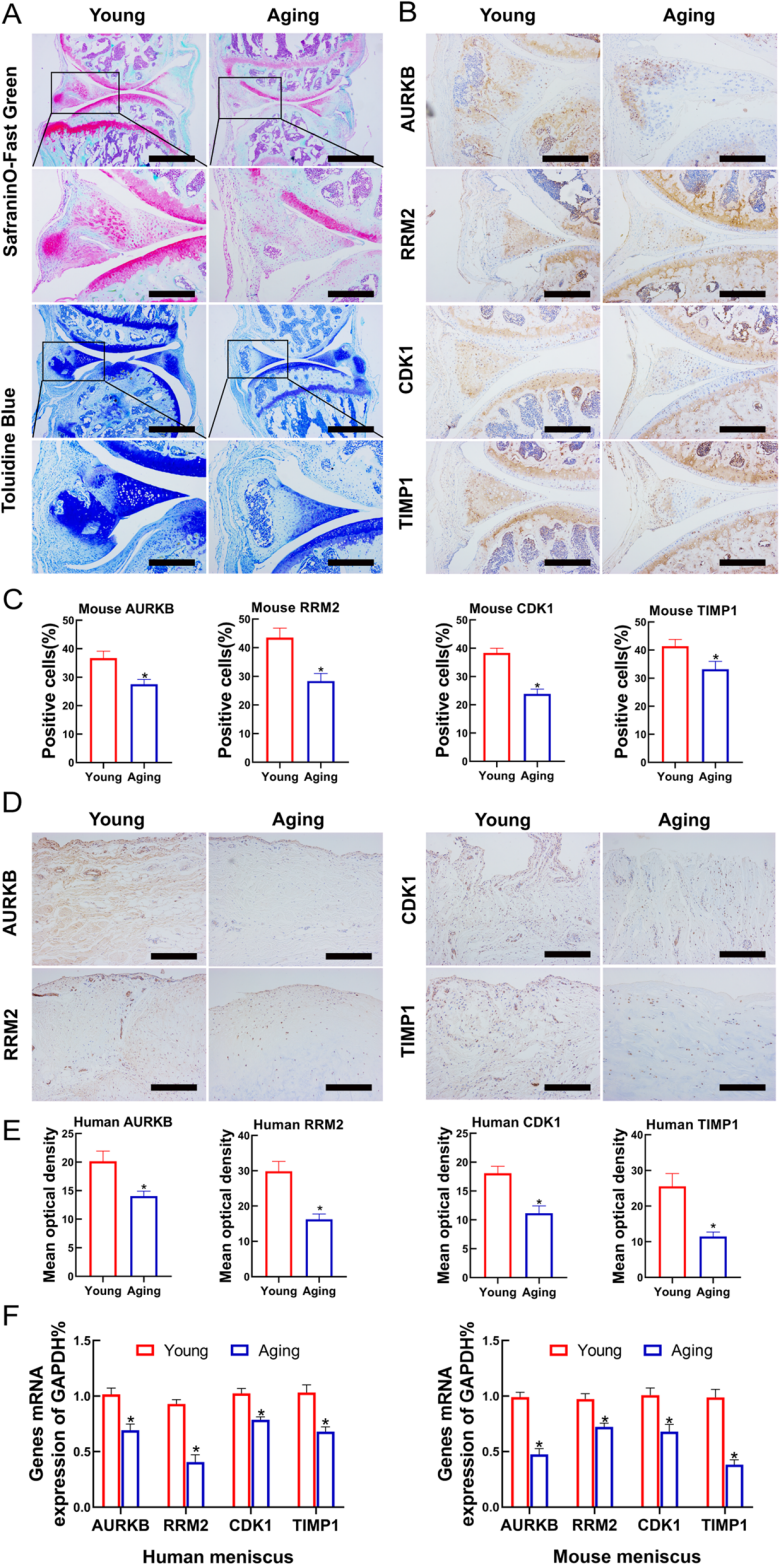
The expression levels of Hub genes in the meniscus tissues. **A** SafraninO-Fast green and Toloniumchloride Blue staining results of meniscus in 18-month-old mice (aging) and 3-month-old mice (young). Scale bar: 250 μm. Scale bar (enlarged): 100 μm. **B** Hub genes immunochemical staining of meniscus in young mice and older mice. Scale bar: 100 μm. **C** Quantification of Hub genes immunostaining (mean optical density). **D** Immunostaining of Hub genes in young and gerontic human menisci. Scale bar: 100 μm. **E** Quantification of Hub genes immunostaining (mean optical density). **F** The mRNA expression levels of Hub genes in human and mouse menisci, respectively. We normalized the gene expression levels to GAPDH. *RRM2* ribonucleotide reductase regulatory subunit M2; *AURKB* aurora kinase B; *CDK1* cyclin dependent kinase 1; *TIMP1* tissue inhibitor of metalloproteinases 1; *GAPDH* Glyceraldehyde 3-phosphate dehydrogenase. *n* = 6 per group, mean ± S.E.M., *P < 0.05 compared with the young group

SafraninO-Fast green and Toloniumchloride Blue staining results of the meniscus of 18-month-old aging mice revealed more severe degenerative histopathological changes than the meniscus in 3-month-old young mice due to decreased collagen fiber content (Fig. 5A). Immunohistochemistry showed that the expression levels of *AURKB, RRM2, CDK1*, and *TIMP1* protein were higher in the meniscus of young mice than that of the senescent mice (Fig. 5B, C). In order to further explore the expression levels of hub genes in senescent meniscus, we applied human meniscus for immunohistochemical detection. The results illustrated that the Hub genes were also poorly expressed in senescent human meniscus (Fig. 5D, E). Moreover, the mRNA levels of *AURKB, RRM2, CDK1*, and *TIMP1* were also decreased both in human and mouse menisci, respectively (Fig. 5F). These founds suggested that the decreased gene expressions of *AURKB, RRM2, CDK1*, and *TIMP1* might be a trigger or indicator that participating in meniscus aging and degeneration.

### Hub genes expression in cultured mouse meniscus cells

**Fig. 6:**
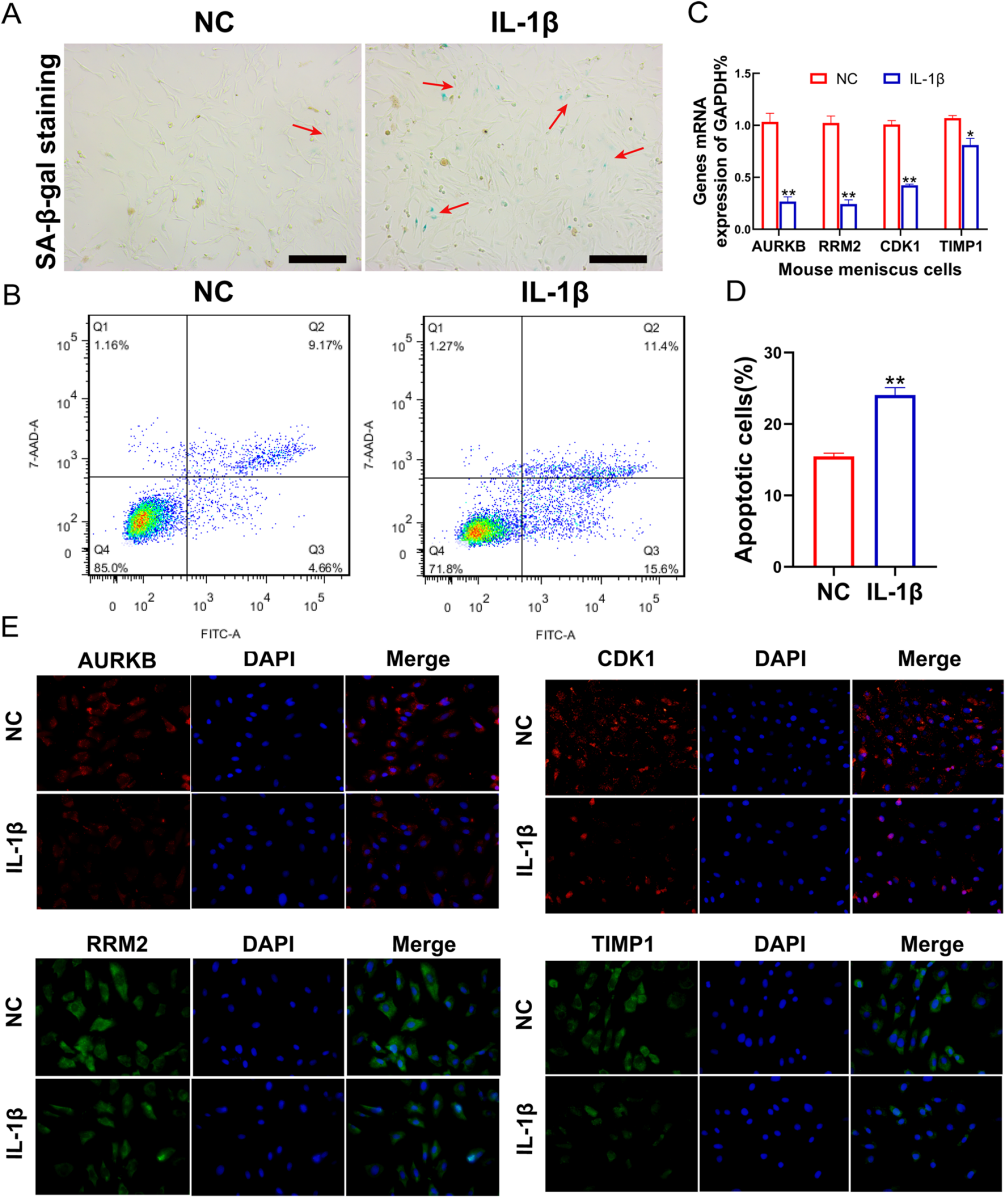
The expressions of Hub genes in the mouse meniscus cells. **A** SA-β-gal staining of mouse senescent meniscus cells induced by IL-1β (10 ng/ml) for 48 h. Scale bar: 500 μm. **B**, **D** Apoptotic analysis detected by Annexin V/7AAD after IL-1β disposition. **C** Detection of the expressions of hub genes in senescent meniscus cells via qRT-PCR. We normalized the gene expression levels to GAPDH. **E** Immunofluorescence analysis of hub genes after treating meniscus cells with10ng/ml IL-1β for 48 h. Scale bars, 50 μm. *RRM2* ribonucleotide reductase regulatory subunit M2; *AURKB* aurora kinase B; *CDK1* cyclin dependent kinase 1; *TIMP1* tissue inhibitor of metalloproteinases 1; *GAPDH* Glyceraldehyde 3-phosphate dehydrogenase. *n* = 6 per group, mean ± S.E.M., *P < 0.05, **P < 0.01 in comparison with the control group

As an additional method to further investigate the expressions of hub genes in senescent meniscus tissues, we treated normal mouse meniscus cells with IL-1β (10 ng/ml) for 48 h to induce degeneration in mouse meniscus cells in vitro. Take into consideration that the overexpression and accumulation of endogenous lysosomal β-galactosidase are one of the characteristics of senile cells, SA-β-gal is often taken as a biomarkers of senile cells [19, 20]. More positive stained cells, the blue ones, were observed in the IL-1β group than the control by the SA-β-gal staining (Fig. 6A). Meanwhile, more apoptotic cells were detected in the IL-1β group than the control by flow cytometry (Fig. 6B, D). In addition, the suppressed mRNAs expressions of *AURKB, RRM2, CDK1,* and *TIMP1* were detected in meniscus cells treated with IL-1β (10 ng/ml) when compared with the control group (Fig. 6C). Then, the immunofluorescence assay further supported that these Hub genes were poor expressions in senescent meniscus cells (Fig. 6E). These consequences further confirmed the decreased *AURKB, RRM2, CDK1,* and *TIMP1* expressions in the senescent meniscus.

## Discussion

The incidence of meniscal injuries is on the rise, which might be partly attributed to the general aging of the population [21]. Previous studies have found that age-related meniscus denaturation induces an increase in fragility of meniscus tissue, thereby increasing the incidence of meniscus tearing in the elderly population [22]. However, the molecular pathologic mechanism of meniscus age-related degeneration is still poorly studied [23]. The high-throughput microarray technology combined with bioinformatics analysis has been widely used to predict the pathogenesis and potential molecular therapeutic targets of many diseases.

In the current study, a microarray dataset GSE45233 containing young and gerontic meniscus samples was obtained and a bioinformatics analysis was completed. In view of the results of GO terms enrichment analysis, we found a relationship between the DEGs and cell senescence. According to the CC analysis results of DEGs, we found that the major changes in the components of the cells were located in the extracellular region. The extracellular domain of meniscus cells mainly refers to the extracellular matrix (ECM), which plays a crucial role in sustaining structural integrity and mechanical properties of meniscus [24]. Histopathological analysis showed that meniscus aging was associated with ECM structure loss [25]. Variations in DEGs related to MF were primarily concentrated in calcium ion binding, and chromatin binding. In fact, imbalance of calcium ion homeostasis and chromatin rearrangement are also often associated with cell senescence and ECM degeneration [26, 27]. Based on the KEGG database, we discovered that the DEGs were principally gathered in the PI3K-Akt signaling pathway, which could act on downstream targets including forkhead box O transcription factors (*FoxO*) and mammalian target of rapamycin (*mTOR*), and also activate gene *P53* to regulate cell growth [28–30]. Herranz et al*.* found that *mTOR* controls senescence secretory by indirectly regulating the expressions of senescence signaling molecules [31]. Furthermore, *FoxO* may play a protective role against meniscus aging by regulating autophagy [32]. Such reports further confirmed our finding.

The PPI network was carried out to predict the connections of protein functions of DEGs, and the common hub genes- *AURKB, CDK1, RRM2,* and *TIMP1-*were screened. *CDK1* is a catalytic subunit of mitogenic promotors and plays a key role in cell cycle control, such as mitosis and G2-M transformation [33]. Saito et al*.* demonstrated that *CDK1* was essential for chondrocyte proliferation and differentiation [34]. *AURKB* is a type of Aurora kinase involved in the regulation of chromosome alignment and separation in mitosis and meiosis [35]. Thus, both *AURKB* and *CDK1* could reflect the meniscus cell cycle to a certain degree. *RRM2* encodes one of two non-identical subunits for ribonucleotide reductase, and catalyzes the formation of deoxyribonucleotides from ribonucleotides [36]. Previous studies have found that the decrease in *RRM2* levels could lead to DNA damage accumulation by inhibiting deoxyribonucleotide triphosphates (dNTPs) expression, thereby mediating cell senescence [37]. *TIMP1*, natural inhibitors of the matrix metalloproteinases (*MMPs*), is a group of peptidases involved in degradation of the extracellular matrix [38]. The balance between matrix degradation and synthesis is impaired during meniscus aging. Since elastolysis or collagenolysis is put down to the joint effect of several members of *MMPs*, *TIMP1* may also play an anti-meniscus aging role to some extent [39]. In the current study, the decrease in the expressions of *AURKB, CDK1, RRM2,* and *TIMP1* was observed in both the human and mouse senescent menisci, as well as meniscus cells treated with exogenous IL-1β. Such finding indicated that both cell cycle arrest and ECM degeneration contribute to the meniscus senescence. In reality, the differences in gene expression in human and mouse menisci in this study have been interpreted as being related to aging. However, they could be also a consequence of meniscus degeneration. Meniscus degeneration is a complex process, which includes cell senescence and death, mechanical and structural damage, extra-cellular matrix degradation, chronic inflammation and nutritional imbalance of the meniscus [40–43] Also, meniscus senescence causatively induces defective extra-cellular matrix synthesis, stimulated tissue inflammation, aberrant cytokine production and tissue degeneration, which further lead to meniscus dysfunction [44, 45]. Thus, we can hardly tell the difference between meniscus senescence and meniscus degeneration in our current study, and the changes observed here might also be results of meniscus degeneration, besides the meniscus senescence [40].

For the five selected key miRNAs targeting the hub genes, there were no relevant studies reporting that they were linked to meniscus before. Further pathways enrichment analysis of these miRNA revealed that these miRNAs were involved in the synthesis of glycosaminoglycans, an important component of the extracellular matrix of the meniscus, of which, the reduction was often accompanied by the occurrence of OA [41]. Cellular senescence is a complex phenotype characterized by two aspects: persistent cell cycle arrest and the production of pro-inflammatory molecules known as senescence associated secretion phenotype (SASP) [42]. Considering that the senescence of articular chondrocytes acting as hyporeplicative cells was mainly driven by SASP factors, we utilized IL-1β (10 ng/ml) to construct an in vitro cell senescence model [43]. Our current study has shown that cell senescence was increased in IL-1β-treated mouse meniscus cells. The decreased expressions of screened hub genes in vitro meniscus cells supported in vivo experiments and further identified these genes were related to cell senescence.

However, our conclusions are only originated from theoretical prediction and models detection, and the role of pivotal genes in meniscus senescence still needs further clinical verification. In addition, our current study is based on a data set from 12 patients, which indeed is a rather small number of patients, although statistical differences were obtained in our study. Whereas, a larger sample size would be certainly more representative, and further studies are needed to add evidence to our current findings. Still, our current study would provide a basis for finding markers of the aging meniscus to a certain extent.

In summary, the most significant 4 hub genes (*RRM2, AURKB, CDK1,* and *TIMP1*) and 5 miRNAs (hsa-miR-6810-5p, hsa-miR-4676-5p, hsa-miR-6877-5p, hsa-miR-8085, and hsa-miR-6133) that regulated such 4 hub genes, were finally identified, which are related to meniscus senescence and could serve as potential biomarkers for age-related meniscus tearing.

## Data Availability

The DNA and RNA sequencing data were downloaded from Genebank database through gene names RRM2 (Gene ID: 6241 and 20135), CDK1(Gene ID: 983 and 12534), AURKB (Gene ID: 9212 and 20877), and TIMP1(Gene ID: 7076 and 21857). Then, the microarray data used in this study could be obtained through GEO ticket number GSE45233 in the GEO database (https://www.ncbi.nlm.nih.gov/gds/?term=GSE45233/).
